# Short read sequence typing (SRST): multi-locus sequence types from short reads

**DOI:** 10.1186/1471-2164-13-338

**Published:** 2012-07-24

**Authors:** Michael Inouye, Thomas C Conway, Justin Zobel, Kathryn E Holt

**Affiliations:** 1 Department of Microbiology and Immunology, The University of Melbourne, Melbourne, Australia; 2 Department of Pathology, The University of Melbourne, Melbourne, Australia; 3 NICTA Victoria Research Laboratory, Department of Computing & Information Systems, The University of Melbourne, Melbourne, Australia

**Keywords:** MLST, Short read, Illumina, Sequence analysis, Plasmid, Chromosome, Microbiology, Bacteria, Population analysis, Outbreak

## Abstract

**Background:**

Multi-locus sequence typing (MLST) has become the gold standard for population analyses of bacterial pathogens. This method focuses on the sequences of a small number of loci (usually seven) to divide the population and is simple, robust and facilitates comparison of results between laboratories and over time. Over the last decade, researchers and population health specialists have invested substantial effort in building up public MLST databases for nearly 100 different bacterial species, and these databases contain a wealth of important information linked to MLST sequence types such as time and place of isolation, host or niche, serotype and even clinical or drug resistance profiles. Recent advances in sequencing technology mean it is increasingly feasible to perform bacterial population analysis at the whole genome level. This offers massive gains in resolving power and genetic profiling compared to MLST, and will eventually replace MLST for bacterial typing and population analysis. However given the wealth of data currently available in MLST databases, it is crucial to maintain backwards compatibility with MLST schemes so that new genome analyses can be understood in their proper historical context.

**Results:**

We present a software tool, SRST, for quick and accurate retrieval of sequence types from short read sets, using inputs easily downloaded from public databases. SRST uses read mapping and an allele assignment score incorporating sequence coverage and variability, to determine the most likely allele at each MLST locus. Analysis of over 3,500 loci in more than 500 publicly accessible Illumina read sets showed SRST to be highly accurate at allele assignment. SRST output is compatible with common analysis tools such as eBURST, Clonal Frame or PhyloViz, allowing easy comparison between novel genome data and MLST data. Alignment, fastq and pileup files can also be generated for novel alleles.

**Conclusions:**

SRST is a novel software tool for accurate assignment of sequence types using short read data. Several uses for the tool are demonstrated, including quality control for high-throughput sequencing projects, plasmid MLST and analysis of genomic data during outbreak investigation. SRST is open-source, requires Python, BWA and SamTools, and is available from http://srst.sourceforge.net.

## Background

Multi-locus sequence typing (MLST) has become the gold standard for the analysis of bacterial populations [1,2]. MLST involves PCR amplification and sequencing of 5–10 loci of ~500 bp in length, with each sequence variant assigned a unique locus variant or allele number. Each unique combination of locus variants is assigned a sequence type (ST), which is then used to denote a precise set of sequences. Public MLST databases are used to store and share information linking DNA sequences to locus variant numbers and sequence types and are available for >85 bacterial species including important human pathogens such as *Staphylococcus aureusHaemophilus influenzae* and *Neisseria* species (see http://pubmlst.org). This format allows quick, simple and direct comparison of bacterial populations analysed in different laboratories and over time. The databases also link individual bacterial isolates to STs, serotypes, sources and other meta-information with public health utility.

Advances in sequencing technology continue to bring down the costs of whole-genome sequencing of bacteria – indeed it is already close to the cost of MLST [3]. Because bacterial genomes are relatively small in size (typically 1–10 Mbp) they can be sequenced in multiplex, allowing the high read depths of e.g. a single lane of Illumina HiSeq to be distributed among up to 96 different samples via the use of individually-tagged libraries [4-7]. Whole-genome sequencing provides a lot more information than MLST and can be used to study microevolution in much finer detail and over small time scales [3-8]. As an assay, whole genome shotgun sequencing can be simpler than MLST, which requires multiple independent PCR and sequencing reactions to be performed for each sample. This will become increasingly true as automation becomes widely used in whole-genome library preparation workflows. Also, while MLST requires the design and purchase of species-specific specific primer sets, whole genome sequencing can be applied to any bacterium, without species primers or even prior knowledge of species. However, while the advantages of whole-genome sequencing over MLST are clear, it is crucial that newly sequenced isolates (or populations of isolates) can be analysed in the context of the vast amount of population data currently stored in MLST databases. MLST can be used to assess the frequency and expansion of particular clones, and most MLST databases store not only ST information for each isolate, but also detailed meta-information such as serotype, host or source, date of collection and in some cases spatial information (see e.g. MLST-maps, http://maps.mlst.net[9]). MLST provides a framework against which new isolate collections can be compared and interpreted, but can only be utilized if the STs of newly sequenced isolates can be accurately derived from whole-genome sequencing data.

The only method currently available to perform MLST allele assignment on short read data is web-based, requiring sequence data to be uploaded to a server and compared to public MLST databases [10]. This poses problems for data security and confidentiality, is unfeasible for the large datasets typically generated in high-throughput multiplex sequencing projects and excludes the use of privately maintained MLST databases. All of these issues are likely to be significant barriers for use in the majority of research or public health laboratories. Furthermore the method depends on *de novo* assembly [10], which limits its sensitivity, particularly for genomes sequenced at low read depth.

Here we present an open-source software tool, SRST, to derive STs from Illumina short read sequence data using a mapping-based approach to maximise sensitivity. SRST can be used together with any public or private MLST scheme and generates output files suitable for comparative analysis with existing MLST datasets, compatible with standard MLST tools such as eBURST [11], ClonalFrame [12] and Phyloviz (http://www.phyloviz.net). In this paper, we introduce the SRST approach and demonstrate its accuracy with real datasets including 534 genomes from four species-specific and four plasmid MLST schemes, and discuss the usefulness of the method for quality control in high-throughput sequencing projects and outbreak investigations.

## Implementation

SRST takes as input (a) locus variant sequences and ST profile definitions, retrieved from a public or private MLST database (such as http://pubmlst.org); (b) sequences flanking each locus, retrieved from an appropriate reference genome sequence using the supplied script; (c) Illumina read data (in fastq format; any number of paired or single-end read files can be processed in a single command). SRST runs on any Linux based computer or cluster (including Mac OS X) and requires the installation of the free packages *BWA* and *SamTools* for alignment functions [13,14]. Full instructions are available at http://srst.sourceforge.net.

Each readset is mapped to each of the possible locus variants *v* (with flanking sequence) and a score *s* is calculated to assess the quality of the match, as follows. Consider a single base position *i* in the mapping of read set *R* to locus variant *v*, in which *n*_*i*_ reads map to position *i,* which has base *v*_*i*_ in *v* and majority-rules consensus (i.e. most prevalent) base *r*_*i*_ in *R*. If *r*_*i*_ ≠ *v*_*i*_ we record a mismatch and rule out *v* as a possible locus variant. Otherwise, we compute the probability that the base call *r*_*i*_ (which matches *v*_*i*_) is erroneous, by calculating Binomial probabilities for the three alternative bases *x*:

(1) $$\mathrm{Pr}\left(\text{X}\geq x\right)=\sum_{j=k}^{n_{i}}\left(\begin{array}{l} n_{t}\\ k \end{array}\right)p^{j}{\left(1-p\right)}^{n_{i}-j}$$

where k = the observed read count for base *x* at position *i*. The probability that a non-consensus nucleotide (which does not match *v*_*i*_) is the best explanation for the coverage at position *i* is the sum of the three probabilities. The probability that a sequence other than *v* is the best explanation for the observed coverage across the whole locus is the sum of these probabilities across all positions *i*. The final score *s* reported is the negative log of this probability, so that higher scores reflect more hits. Note this treats the probabilities at each position as independent, which they are not. However the assumption is conservative, and only results in non-trivial over-estimation of the probability in cases where the score will be below a threshold of acceptance in any case.

For each readset and locus, the highest scoring variant, with zero mismatches and passing a user-settable cut-off value, is assigned. If all loci are assigned for a particular readset and the combination of variants is a known ST, this ST will be returned; if a novel combination of locus variants is detected this will be assigned a novel ST. If any loci are not confidently assigned, no ST can be called.

The main output is a file specifying the locus variants and STs of all input datasets, suitable for analysis with common MLST tools such as eBURST or Phyloviz.

A log file is also generated, detailing scores and coverage statistics for each locus and readset. Where an exact match to a known allele cannot be found, the closest allele (and number of mismatching bases) is reported and the closest ST is determined; these results are flagged so as to be distinguished from precise ST assignments.

Optionally, verbose output can be switched on in order to retain full sequence information for novel alleles, including the alignment (bam format), pileup (pileup format) and consensus sequence (fastq format) obtained from mapping to the closest-matching locus variant. See Additional file 1 for an example fastq generated by SRST. This is intended to facilitate investigation of novel alleles, in which case visual inspection of alignments or pileups is recommended. Some MLST databases may also accept fastq or bam files for submission of novel alleles.

## Results and Discussion

### Accuracy in calling sequence types

**Figure 1:**
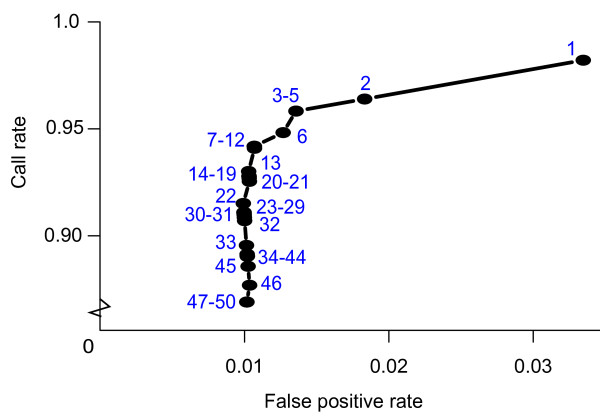
**Score threshold determination for SRST.** Each data point indicates the call rate (y-axis) and false positive rate (x-axis) for a given cut-off score, labeled in blue. False positive rate = proportion of loci with incorrect variant calls, call rate = proportion of loci for which a variant could be confidently assigned, total loci = 2,387 (341 samples, 3 species)

To determine a suitable cut-off score and test the accuracy of SRST, we utilized three publicly available datasets each representing a different group of bacteria – *Streptococcus pneumoniae*[6,15], *Staphylococcus aureus*[4,16] and *Salmonella bongori*[17,18]. All short read, MLST and reference data were downloaded from public databases (Table 1). We ran SRST on each read set (N = 341 genomes, 2,387 loci) and examined the sensitivity (call rate; i.e. the proportion of loci for which a variant could be confidently assigned) and specificity (false positive rate; i.e the proportion of loci with incorrect variant calls) obtained using different cut-off scores. As Figure 1 shows, best results were obtained with a cut-off score between 7 and 11. We therefore set the default cut-off score for SRST to 10, and use this cut-off for all subsequent analyses reported below. In the few cases where an allele could not be confidently assigned using a cut-off score of 10, the expected allele and ST were still correctly identified by SRST as the most likely result.

**Table 1:** High throughput read sets analyzed in this study

**Species**	**N**	**Read type**	**Accession (reads)**	**MLST database**	**Reference genome**
*Staphylococcus aureus*[4]	67	37 bp SE	ERP000070	http://saureus.mlst.net/	NC_002952.2
*Salmonella bongori*[17]	18	54 bp PE	ERP000328	http://mlst.ucc.ie/mlst/	NC_011900.1
*Streptococcus pneumoniae*[6]	256	54 bp PE	ERP000139	http://spneumoniae.mlst.net/	NC_011149.1
*Shigella sonnei*[19]	188	54 bp PE	ERP000182	http://mlst.ucc.ie/mlst/	NC_000913.2

PE = paired end sequencing, SE = single end sequencing.

### Quality control in high-throughput sequencing projects

The most obvious application of SRST is to assign STs to novel isolates whose STs are unknown. However we were also interested to use SRST for quality control in large-scale sequencing studies of bacterial clones, to allow early detection and identification of read sets that should be excluded from comparative analysis of the clonal group of interest. To demonstrate the utility of SRST for this purpose, we used it to analyse a set of *Shigella sonnei* genomes sequenced using paired-end multiplex Illumina GAII (Table 1) [19]. Note *Shigella* species are actually sublineages of *E. coli*[20], hence the *E. coli* MLST scheme [21] is used to investigate *Shigella*.

A total of 188 *Shigella* data sets had sufficient mean read depth (>10x) to analyse and 170 (90 %) of these matched a known ST with SRST scores ≥10 for all loci. The other 18 samples comprised (i) 15 with ≥1 locus scoring <10 but identified as a known *S. sonnei* allele and (ii) 3 with novel locus variants (each having one locus for which the highest scoring match (scores 20–160) differed from a known *S. sonnei* allele by one mismatching base). Hence all 18 were recognizable as single-locus variants of known *S. sonnei* STs, confirming their suitability for inclusion in a *S. sonnei* study. Consensus sequences and quality scores for the novel alleles, generated using SRST’s verbose option, are given in Additional file 1. Of the 170 STs assigned with score ≥10, 166 matched known *S. sonnei* STs (ST152, ST1502, ST1504, ST1505). Four matched those of other, non-sonnei, *Shigella* (*S. flexneri*, ST245; *S. boydii,* ST243, ST1025), indicating they were erroneously included in the *S. sonnei* isolate collection and should be excluded from the intended analysis of *S. sonnei*. The species status of these four isolates, identified by SRST, was confirmed by serotyping of the original isolates, and comparison of the read sets to reference genomes of *S. flexneri* and *S. boydii*. Hence SRST could successfully detect and identify outliers for removal. In contrast, allele assignment by blastn search of *de novo* assembled contigs (assembled using Velvet 1.0.13 and Velvet Optimiser 2.1.7) succeeded for only 60 % of the *Shigella* read sets.

### Plasmid MLST

**Figure 2:**
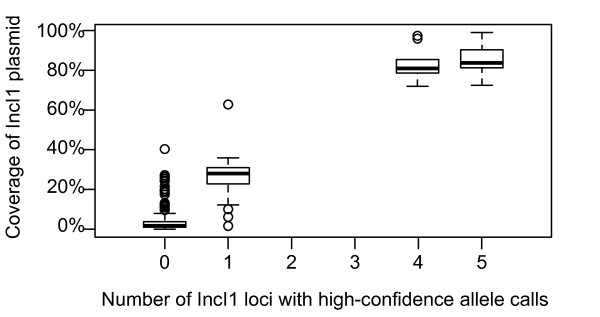
**Use of SRST to detect plasmids.** X-axis indicates the number of IncI1 plasmid MLST loci with a high-confidence allele assignment (score > 10) using SRST; y-axis indicates the proportion of the IncI1 ST16 reference plasmid, pEK204, covered by each read set

There are currently MLST schemes available for four types of plasmid – IncI1 [22], IncN [23], IncHI1 [24] and IncHI2 [25] (http://pubmlst.org/plasmid/). To test the accuracy of SRST for plasmid MLST, we used it to detect and assign 5-locus STs to IncI1 plasmids in the *S. sonnei* dataset. Since we do not have traditional plasmid MLST sequences available as a control, we mapped the reads to the reference sequence for IncI1 ST16 plasmid pEK204 (NC_013120) [26] and used the proportion of the plasmid covered by each read set as a measure of the real presence of IncI1 plasmids in the data (e.g. 90 % coverage of the reference plasmid would indicate an IncI1 plasmid is present; 10 % coverage of the reference plasmid would indicate no IncI1 plasmid is present). As Figure 2 shows, there was a strong correlation between the number of IncI1 plasmid MLST loci that could be assigned with confidence by SRST, and the coverage of the IncI1 reference plasmid, indicating that SRST is useful for screening for the presence of specific plasmid types. A total of eight read sets were assigned the same IncI1 ST (16) as pEK204; their coverage of pEK204 ranged from 94.2 %-99.0 % (mean 96.9 %), while the highest coverage of pEK204 in other read sets was 92.3 % (assigned to ST37). This suggests that SRST’s assignment of plasmid STs is as accurate as that for chromosomal STs.

**Figure 3:**
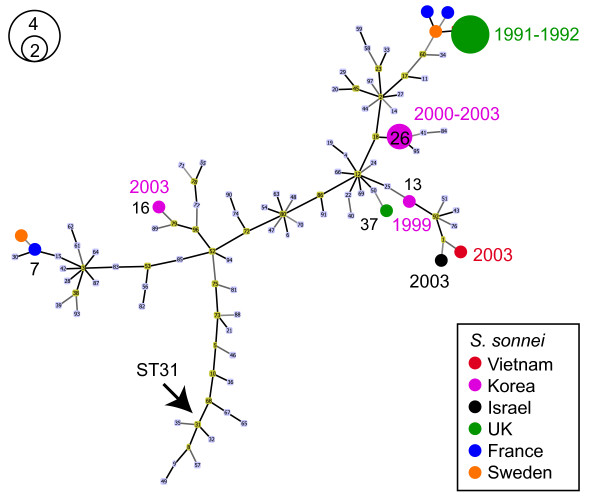
**IncI1 plasmid STs detected among*S. sonnei.*** Minimum spanning tree of all IncI1 STs present in the IncI1 plasmid MLST database as at March 13, 2012, generated using Phyloviz (http://www.phyloviz.net). STs detected among *S. sonnei* read sets are highlighted; node colour indicates the country of origin according to the legend provided (bottom-right); node size indicates the number of isolates according to the inset circle legend (top-left). Nodes representing known STs are labeled in black with the ST number (7, 13, 16, 26, 27, 55), unlabeled nodes are novel STs (novel combinations of known alleles). Dates of isolation are indicated for larger groups (coloured text). Arrow indicates the position of ST31, identified in isolates of *E. coli* O104:H4 associated with an outbreak in Germany in 2011

High-confidence IncI1 plasmid STs were assigned to a total of 26 *S. sonnei*. As Figure 3 shows, these represent a variety of very distinct IncI1 plasmid types, indicative of multiple transfers of divergent IncI1 plasmids into *S. sonnei*. The plasmid STs clustered geographically and, within geographic regions, temporally (Figure 3), suggesting there have been several, highly localized, transfers of distinct IncI1 plasmids into the global *S. sonnei* population.

### Outbreak analysis

Chromosomal and plasmid MLST can provide useful insights into bacterial pathogen outbreaks. SRST allows these insights to be rapidly extracted from whole-genome shotgun sequencing, which can be performed without prior knowledge of the species and with no need for PCR with species-specific MLST primers. To illustrate this, we utilised five Illumina short read data sets from the outbreak of *E. coli* O104:H4 causing hemolytic uremic syndrome in Germany in 2011 [27,28] (accessions SRP000285, SRP008003, SRP008032-36, SRP007327). The data was generated in two different sites (BGI, China and Broad Institute, US). We used SRST to screen the outbreak data using the *E. coli* MLST database to identify the chromosomal ST and all publicly available plasmid MLST databases to identify IncI1, IncN, IncHI1 or IncHI2 plasmids (see above). Details of the datasets and results are provided in Table 2.

**Table 2:** **Chromosomal and plasmid analysis of*E. coli*outbreak strains**

**Strain**	***E. coli*ST (score)**	**IncI1 ST (score)**	**Accession**	**Reference**
C227-11	ST678 (24660)	ST31 (2320)	SRP000285	[27]
C236-11	ST678 (27131)	ST31 (2723)	SRP008003	[27]
11-3677	ST678 (20353)	ST31 (11286)	SRP008034	[27]
11-3798	ST678 (10400)	ST31 (6848)	SRP008035	[27]
TY-2482	ST678 (1042)	ST31 (326.5)	SRP007327	[28]

The SRST ST calls and scores are indicated for the chromosome (*E. coli* scheme) and plasmid (IncI1 scheme). No other plasmids with MLST schemes available (IncN, IncHI1, IncHI2) were detected using SRST

SRST correctly identified the chromosomal ST of all five outbreak isolates as *E. coli* ST678, which matches that reported using traditional MLST approaches [29]. The closest available finished reference genome sequence to the *E. coli* outbreak strain, Ec55989, shares this ST, and has formed the reference for phylogenetic and gene content analyses of the German outbreak in all published studies [27-30]. This illustrates the utility of SRST to rapidly identify the most suitable reference sequence for whole-genome analysis during an outbreak. At the time of the German outbreak, Ec55989 had not been entered into the *E. coli* MLST database and extensive read mapping to all available *E. coli* sequences (approximately N = 60 at the time) was required to identify the most suitable reference [28], however assuming all available reference sequences are entered into the relevant MLST databases, this could be achieved much more quickly and easily in future using SRST.

SRST also correctly identified the presence of an IncI1 plasmid of ST31 in each of the *E. coli* O104:H4 outbreak isolates (arrow in Figure 3). The outbreak strain’s antibiotic resistance plasmid was previously confirmed as IncI1 ST31 using traditional plasmid MLST (present in the IncI1 database as CTX-I1-O104:H4, see http://pubmlst.org/plasmid/). As Figure 3 shows, this plasmid is quite divergent from any we detected in the *S. sonnei* data. The IncI1 plasmid MLST database shows IncI1 ST31 plasmids have previously been identified in a variety of other *E. coli* hosts circulating in both humans and animals, often containing extended spectrum beta-lactamase CTX-M genes similar to that encoded in the outbreak isolates’ IncI1 plasmids (see IncI1 database at http://pubmlst.org/plasmid/). No IncN, IncHI1 or IncHI2 plasmids were identified by SRST, consistent with published reports of the outbreak genomes [27-30].

### Other potential applications

As SRST is database driven it could be used for other sequence typing tasks beyond MLST, provided appropriate databases are used as input. For example, it could be used to annotate drug resistance genes and alleles. Used in conjunction with the recent ribosomal MLST database [31], SRST could potentially be used for species designation of novel isolates. These applications could be useful in outbreak analysis, strain identification, surveillance, studies of mechanisms and transfer of drug resistance, and a variety of other public health and research applications.

## Conclusions

SRST uses read mapping to assign sequence types to novel bacterial genomic sequence data, which offers several advantages over traditional MLST using PCR and Sanger sequencing. SRST is accurate and sensitive at allele assignment and can identify and retrieve novel allele sequences for further investigation. Being mapping-based it is more sensitive than assembly-based allele assignment for short read data sets, and can be run locally without reliance on web-based services or data uploads. SRST can be used in a variety of contexts, including simple allele assignment to novel data sets, quality control in batch sequencing projects, outbreak investigation, plasmid MLST and potentially in any scenario where database-driven sequence typing is required.

## Availability and requirements

**Project name:** SRST (Short Read Sequence Typing)

**Project home page:**http://srst.sourceforge.net/

**Operating system(s):** Linux/Mac

**Requirements:** samtools 0.1.8, BWA 0.5.7 (open-source)

**Programming language:** Python 2.6.4

**License:** BSD

**Any restrictions to use by non-academics:** No

## Competing interests

The authors’ declare that they have no competing interests.

## Authors’ contributions

All authors contributed to methods design, editing and approved the final manuscript. TCC also wrote and tested code. MI performed software testing and data analysis. KEH wrote and tested code, sourced data, performed data analysis and drafted the manuscript. All authors read and approved the final manuscript.

## Supplementary Material

Additional file 1 **Example of novel allele output using the verbose option.** Consensus sequences and quality scores for three novel *Shigella sonnei* alleles identified using SRST. (TXT 3 kb) Click here for file
